# Long-term respiratory symptoms following oesophageal atresia

**DOI:** 10.1111/j.1651-2227.2011.02279.x

**Published:** 2011-09

**Authors:** Vladimir Gatzinsky, Linus Jönsson, Linda Ekerljung, Lars-Göran Friberg, Göran Wennergren

**Affiliations:** 1 Department of Paediatric Surgery, University of GothenburgGothenburg, Sweden; 2 Krefting Research Centre, University of GothenburgGothenburg, Sweden; 3 Department of Paediatrics, University of GothenburgGothenburg, Sweden

**Keywords:** Asthma, Oesophageal atresia, Outcome, Respiratory symptoms, Wheezing

## Abstract

**Background:**

Oesophageal atresia (OA) is a congenital malformation that can lead to persistent respiratory symptoms in adulthood.

**Aim:**

To describe the prevalence of respiratory symptoms in adulthood in a population-based study of patients with repaired OA and to compare this with the prevalence in the general population.

**Methods:**

Of 80 patients operated for OA in Gothenburg in 1968–1983, 79 were located. The patients received a questionnaire on respiratory symptoms. Controls were 4979 gender- and age-matched subjects who answered the same questions.

**Results:**

The questionnaire was answered by 73 of 79 (92%) patients. Physician-diagnosed asthma was reported by 30% in the OA group vs 10% in the control group (OR 4.1; 95% CI 2.4–6.8), and recurrent wheeze in 29% vs 5.5% (OR 6.9; 4.1–11.6). Also wheeze during the last year, asthma medication, a long-standing cough, cough with sputum production and chronic bronchitis were significantly more common among the patients with OA. In contrast, there was no significant difference regarding risk factors for asthma. The prevalence of respiratory symptoms did not appear to decrease with age.

**Conclusion:**

A high prevalence of respiratory symptoms remains among adult patients with repaired OA. Many of the patients had an asthma diagnosis. However, asthma heredity or allergic rhinitis was not overrepresented.

## Introduction

Oesophageal atresia (OA) is a congenital malformation that was previously fatal. In the last few decades, OA has become a condition with a survival rate exceeding 90% (1). The treatment is surgical reconstruction or substitution of the oesophagus.

As a growing number of survivors are now reaching adulthood, there is considerable interest in the long-term outcome in terms of morbidity and quality of life (QoL). We know that respiratory symptoms, dysphagia and gastro-oesophageal reflux (GER) are overrepresented when the subjects reach adulthood (2,3).

There are many factors that could contribute to the respiratory symptoms after the repair of OA. A possible association between GER and asthma is well known (4). Among patients with OA, abnormal airway epithelium and tracheomalacia could also contribute to the symptoms (1,2).

Although it has been reported that the respiratory symptoms become less frequent with time (2), the problems continue into adulthood in many subjects (5). However, there is still a shortage of population-based studies in which the respiratory symptoms during adulthood are compared with the prevalence in the general population. There is therefore a need for more information on the long-term outcome in this patient group.

The aim of this population-based study was to describe the occurrence of respiratory symptoms in adulthood among patients undergoing surgery for OA, compared with the presence of the symptoms in the general population.

## Material and methods

### Patients

Between 1968 and 1983, 110 consecutive patients with OA were identified and treated at the Children's Hospital in Gothenburg. The patients and their characteristics have previously been described by Sillén et al. (6).

**Key notes**

- A high prevalence of respiratory symptoms including asthma-like symptoms, wheeze and cough remains in adulthood in patients with repaired oesophageal atresia.
- Many of the oesophageal atresia patients had an asthma diagnosis. However, the high prevalence of allergic rhinitis and a family history of asthma, usually seen in asthma, were not seen.
- In this study, the prevalence of respiratory symptoms did not appear to decrease with age.

The catchment area is the region of western Sweden. All children born with OA within this area are referred to the Department of Paediatric Surgery at the Children's Hospital in Gothenburg. The study is therefore population based. The region has 1.5 million inhabitants, one-sixth of the population of Sweden. The largest city in the region is Gothenburg, with 500 000 inhabitants.

Of 80 survivors, 79 (42 men and 37 women) were located through the Swedish Population Register. Their hospital records were reviewed for clinical data.

Of the 79 patients, 69 (87%) had OA with a distal tracheo-oesophageal fistula (TOF), three had OA without a TOF (pure OA), five had a TOF without any OA, one had OA with a proximal fistula and one had OA with double TOFs (7).

Associated malformations were present in 28 (35%) of the patients (Table 1). A diagnosis of VACTERL (vertebral, anorectal, cardiac, tracheo-oesophageal, renal and limb) association (defined as the presence of three or more components) was made in seven patients (9%).

**Table 1:** Associated malformations in the patients with oesophageal atresia (n = 79)

Associated malformations	Number of patients (%)
Gastrointestinal	9 (11.4)
Musculoskeletal	9 (11.4)
Congenital heart disease	7 (8.9)
Urogenital	5 (6.3)
Face	3 (3.8)
CNS	2 (2.5)
Chromosomal aberration	1 (1.3)

CNS = central nervous system.

### Questionnaires

In the spring of 2008, the located 79 patients were contacted with a letter describing the study. The patients were asked whether they were willing to participate. If no answer was received, the patients were reminded by telephone and by letter. The patients were offered to make contact by telephone if they had any questions about the study. The study is part of a larger investigation of the long-term outcome in adults with repaired OA. Results regarding their QoL and symptoms of dysphagia have been reported in a previous article (7).

The patients that agreed to participate received a questionnaire with 33 questions based on the Swedish OLIN (Obstruktiv Lungsjukdom i Norrbotten, Obstructive Lung Disease in Northern Sweden) questionnaire (8). The OLIN questions have been used in several studies of obstructive lung disease in Northern Europe. The questionnaire contained questions on obstructive respiratory disease, respiratory symptoms, rhinitis and possible risk factors for disease, such as smoking and a family history of asthma or allergy. The patients were also asked about medication, weight, height and possible other diseases.

As controls, 4979 gender- and age-matched subjects from the same geographical region were used. These subjects were obtained from the West Sweden Asthma Study where data collection was also performed in 2008 (9). The West Sweden Asthma Study also uses questions from the OLIN study. As a result, the questions in the OA questionnaire were the same as in the West Sweden Asthma Study from which appropriate data were extracted. To make our study comparable to previous studies on respiratory symptoms in patients with repaired OA, we focused on 12 questions related to respiratory symptoms, allergy and smoking.

### Questions

*Physician-diagnosed asthma:*‘Have you been diagnosed as having asthma by a doctor’? *Asthma medication:*‘Do you currently use asthma medicine (permanently or as needed)’?

*Wheezing last 12 months: ‘*Have you had whistling or wheezing in the chest on any occasion during the last 12 months’?

*Recurrent wheeze: ‘*Do you usually have wheezing or whistling in your chest when you breathe’?

*Long-standing cough: ‘*Have you had a persistent cough during the last few years’?

*Cough with sputum production: ‘*Do you usually produce phlegm when you cough or do you have phlegm in your chest which is difficult to bring up’?

*Chronic bronchitis: ‘*Have you ever had a cough with phlegm for 3 months or more a year for at least 2 years’?

*Allergic rhinitis:*‘Have you ever had allergic eye or nose problems (hay fever)’?

*Family history of asthma:*‘Has any of your parents or siblings ever had asthma’?

*Family history of allergic rhinitis:*‘Has any of your parents or siblings ever had allergic eye or nose problems (hay fever)’?

*Family history of chronic bronchitis or emphysema: ‘*Has any of your parents or siblings ever had chronic bronchitis or emphysema’?

*Smokers* reported smoking during the year preceding the survey. *Ex-smokers* reported having stopped smoking at least 12 months prior to the survey.

### Statistical methods

For comparisons between the two groups, Fisher's exact test was used for dichotomous variables. Odds ratios (OR) with 95% confidence intervals (CI) were calculated from 2 × 2 contingency tables using standard methods.

### Ethical approval

The study was approved by the ethics committee at the University of Gothenburg.

## Results

Of the 79 patients, 73 (92%) completed the questionnaire. The mean age was 31.7 years for the participants (median 31; range 25–40) and 32.6 years for the controls (33; 25–40). The mean weight and height of the women with repaired OA was 65 kg (range 43–130) and 165 cm (range 158–173), and for men it was 77.5 kg (58–120) and 178 cm (164-190). The mean body mass index (BMI) for the group with repaired OA was 24 (range 17.6–50.8). Three patients did not provide complete information on their weight, and one patient did not mention his height.

### Concomitant diagnoses

Three patients reported having a psychiatric diagnosis. Two patients had an inflammatory bowel disease. One patient, born with a ventricular septum defect, had a pacemaker. One patient had hepatitis and one reported having gout.

### Respiratory symptoms

The prevalence of respiratory symptoms is presented in Table 2. Respiratory symptoms were consistently more common among patients with OA than controls. Physician-diagnosed asthma was reported by 30% in the OA group vs 10% in the control group (p < 0.001). Asthma medication was used by 24% of the patients with repaired OA vs 9% of the controls (p < 0.001). However, only 7% in the OA group used asthma medication on a regular basis. These five patients all used combination therapy with an inhaled corticosteroid plus a long-acting bronchodilator.

**Table 2:** Prevalence of various respiratory symptoms in patients with repaired oesophageal atresia and controls

Variable	OA, n (%)	Controls, n (%)	OR (95% CI)
Physician-diagnosed asthma	22 (30.1)	477 (9.6)	4.1 (2.4–6.8)
Asthma medication	17 (23.9)	432 (8.7)	3.3 (1.9–5.8)
Wheezing last 12 months	32 (44.4)	825 (16.6)	4.0 (2.5–6.4)
Recurrent wheeze	21 (28.8)	275 (5.5)	6.9 (4.1–11.6)
Long-standing cough	22 (30.1)	573 (11.5)	3.3 (2.0–5.5)
Cough with sputum production	25 (34.2)	633 (12.7)	3.6 (2.2–5.8)
Chronic bronchitis	10 (13.7)	321 (6.4)	2.3 (1.2–4.5)

OA = oesophageal atresia, OR = odds ratio, 95% CI = 95% confidence interval.

Also wheeze during the last year, recurrent wheeze, a long-standing cough, cough with sputum production and chronic bronchitis were significantly more common among the patients with repaired OA than among the controls.

The OR for physician-diagnosed asthma among the patients with repaired OA compared with the control group was 4.1. Similarly, the OR for using asthma medication was 3.3, while the OR for recurrent wheeze was 6.9.

In contrast, there was no statistically significant difference between the OA and control group regarding reported allergic rhinitis (current or previous), 41% vs 32% (p = 0.16).

### Heredity and smoking

There was no statistically significant difference in terms of a family history (parents or siblings) of asthma, allergic rhinitis or chronic bronchitis between the patients with repaired OA and the controls. Nor was there any statistically significant difference in the prevalence of smokers or ex-smokers between the groups (Table 3).

**Table 3:** Prevalence of heredity and smoking in patients with repaired oesophageal atresia and controls

Variable	OA, n (%)	Controls, n (%)	p-value
Family history of asthma	18 (25.4)	943 (19.6)	0.2902
Family history of allergic rhinitis	23 (32.9)	1874 (38.8)	0.3762
Family history of chronic bronchitis or emphysema	4 (5.7)	275 (5.8)	1.000
Smoker	14 (19.2)	677 (14.0)	0.2720
Ex-smoker (stopped >1 year ago)	15 (25.9)	810 (18.9)	0.2422

OA = oesophageal atresia.

## Discussion

The main finding in this study is the high prevalence of respiratory symptoms in adulthood in the OA group compared with the controls, including a diagnosis of asthma and the use of asthma medication.

To our knowledge, there is only one previous population-based study of operated OA followed to adulthood, and our results of a high rate of respiratory morbidity in the OA group are in agreement with the results of this recent Finnish study (5).

Wheezing is one of the main respiratory symptoms following repaired oesophageal atresia (1,2). This was clearly seen in the present follow-up where the prevalence of wheeze and asthma diagnosis (44% and 30%) was even higher than in the Sistonen study (37% and 16%) (5).

In the Sistonen study, the occurrence of respiratory symptoms among the adult patients with repaired OA was lower than what has been reported for children and adolescents with OA (5), suggesting an improvement with age (2,10). Regrettably, we do not have data on the previous prevalence of respiratory symptoms in our OA cohort. However, when compared with studies performed in childhood or adolescence (11), our follow-up data do not indicate a reduced prevalence with age. Chetcuti and Phelan reported that the prevalence of a persistent cough declined from 32% in patients at preschool age to 9% among patients over 15 years (2). This can be compared with our high prevalence of a long-standing cough. However, the possibility exists that the severity had decreased, despite a high percentage reporting still having symptoms.

The prevalence of asthma among patients with OA varies between 12% and 29% in different studies (5,10–15). One question is whether the diagnostic term ‘asthma’ is correctly used in this patient group. This question cannot be answered in a questionnaire-based study, but some observations may be of interest in this context. Even though physician-diagnosed asthma was significantly higher in the OA group, there was no over-representation of rhinitis or a family history of asthma which usually is the case in subjects with asthma (9). This could lend support to a speculation that some of the patients with OA have asthma-like symptoms rather than asthma, a speculation supported by observations in other studies. For example, Agrawal et al. (14) examined the effect of bronchodilators in 12 children with repaired OA. Of 4 patients having a diagnosis of asthma, only one responded to salbutamol. In adolescents with repaired OA, Malmström et al. (11) did not find any significant association between bronchial responsiveness to histamine and doctor-diagnosed asthma. Nor did bronchial biopsies demonstrate histological findings typical of asthma. Furthermore, there was no significant correlation between exhaled nitric oxide (NO) levels, which are expected to increase in eosinophil airway inflammation, and the respiratory symptoms of patients with OA. Normally, asthma is associated with bronchial hyper-responsiveness (16), but although many patients with repaired OA have bronchial hyper-responsiveness (17), few studies have demonstrated a correlation with current respiratory symptoms (5). Other studies have failed to find a significant correlation between the respiratory symptoms in patients with OA and a positive airway challenge (12,18).

Taken together, this suggests that caution should be advised when deciding whether an OA patient with asthma-like symptoms actually has asthma.

The significant increase in the risk of a long-standing cough and a cough with sputum production among the OA subjects could not be explained by smoking because there was no statistical difference in smoking prevalence between the OA group and the controls.

The majority of the patients underwent surgery for the most common type of OA, that is, OA with a distal TOF. The other four types of OA represented only small numbers (7), and for this reason, we did not feel that it was reliable to analyse whether or not the respiratory symptoms were correlated with the type of atresia.

The weaknesses of this study are those inherent to all questionnaire-based studies regarding the validity of answers. Another weakness is the fact that we do not have lung function measurements of the study subjects. Such measurements are planned in the next phase of this study.

The strengths of the study are that it is population based and that it has a high response rate, over 90%. Another strength is that the comparisons were made with a large control group that is representative of the general population (19).

## Conclusion

To summarize, the high prevalence of respiratory symptoms remains in adulthood in patients with repaired OA. Many of the patients have asthma-like symptoms and an asthma diagnosis. However, the high prevalence of allergic rhinitis and a family history of asthma that is usually seen in asthma were not seen in the OA group.
